# Noncoding RNAs in Health and Disease

**DOI:** 10.1155/2018/9135073

**Published:** 2018-01-22

**Authors:** Davide Barbagallo, Gaetano Vittone, Massimo Romani, Michele Purrello

**Affiliations:** ^1^Section of Biology and Genetics Giovanni Sichel, Department of Biomedical and Biotechnological Sciences, University of Catania, Catania, Italy; ^2^Section of Philosophical, Psychological, Pedagogical and Social Sciences, Department of Humanities, University of Catania, Catania, Italy; ^3^Laboratory of Tumor Epigenetics, IRCCS AOU San Martino-IST, Genova, Italy

Genome and RNA sequencing have reliably demonstrated that up to 90% of the human genome may be transcribed. According to actual estimates, the *proteome genome* (i.e., all genomic sequences involved in mRNA synthesis) occupies only up to 4% of the human genome [1]. The remaining part, which may be provisionally named as *noncoding RNA genome* (*ncRNA genome*), comprises genes encoding a plethora of structurally and functionally different RNAs other than mRNAs: since most of these apparently do not code for proteins, they have been labelled noncoding RNAs (ncRNAs) [2]. These data have determined a radical paradigm shift in biomolecular medicine and modified our vision of genome structure and functions. They also changed objectives and perspectives of genomic research. As reminded above, the *ncRNA genome* very likely occupies a very large molecular space; accordingly, it seems logical to hypothesize that the molecular explanation of important biopathological phenomena (i.e., normal development and differentiation together with their pathological alterations) is to be found in these regions. It is interesting to remark that they were once deemed to be devoid of any biological function and inappropriately labeled as *garbage DNA*. Different parameters are actually applied to classify and analyze ncRNAs: (1) molecular length and structure [e.g., microRNAs (miRNAs), long ncRNAs (lncRNAs), and circular RNAs (circRNAs)]; (2) mode of expression (housekeeping ncRNAs versus cell type-specific ncRNAs); (3) molecular mechanism of action (miRNAs are mechanically the best characterized ncRNAs); (4) organism where they are expressed (eukaryotes, prokaryotes, and Archaea). Concerning this last point, ncRNAs have been detected in organisms at all evolutionary levels, including bacteria: this confirms their high functional biomolecular importance within living organisms. This special issue comprises 5 research and 6 review articles, which describe several aspects of the molecular structure and functions of ncRNAs (miRNAs, lncRNAs, and circRNAs), both in physiology and in pathology. Identification and characterization of structure and function of new RNA molecules are easier and faster to obtain than in the past thanks to the recent progress in high-throughput molecular biology techniques and bioinformatic analysis, especially applied to next-generation sequencing (NGS). Among ncRNAs, miRNAs are the most known and well characterized: they belong to the class of small ncRNAs and act as negative regulators of gene expression at posttranscriptional level [3–6]. Each of them is estimated to regulate the expression of up to 200 different target mRNAs; accordingly, miRNAs occupy a leading position within molecular cell networks and their function is critical in regulating both physiological and pathological processes (e.g., cancer and neurodegenerative and neuropsychiatric diseases). Research in the field of miRNAs, and ncRNAs in general, ranges from investigation of pathways that are involved in their use as therapeutic agents and as diagnostic and prognostic biomarkers. All these aspects are dealt with within this special issue. I. Grossi and colleagues review the characterized functions of miR-193a-3p, both as physiological negative regulator of cell cycle progression in several cell types (i.e., endothelial colony forming cells, myofibers, and uterine epithelial cells) and as tumour suppressor downregulated in several cancers. The same authors suggest an in-depth analysis aimed to unravel the role of miR-193a-3p in Parkinson's disease or schizophrenia. Study of physiological processes as ageing can suggest how a specific pathway is perturbed in pathological condition: this is the case of the paper by P. Balaskas and colleagues who suggest a battery of miRNAs for future functional studies in osteoarthritis based on their analysis of differential expression of miRNAs in joints or cartilage during ageing. B. Banelli and colleagues review the involvement of miRNAs in the pathogenesis of glioblastoma multiforme (GBM), encompassing the interplay between miRNAs and epigenetic cell networks as well as the proposed role of miRNAs as candidates for innovative therapies in GBM. Biomolecular effects of miRNAs are strictly related to cell context; for instance, a specific miRNA can act as a tumour suppressor or oncogene in different cancers, depending on the targets it recognizes in a specific biomolecular context. A. Izzo and colleagues deal with this issue in a paper on the involvement of altered miRNAs expression in heart defects of Down syndrome fetuses. M. Rizzo et al. describe the *targetome* of miR-28-5p in the prostate cancer cell context. Identification of molecular mechanisms and pathways regulated by miRNAs is critical to expand knowledge on cell physiological processes or to determine onset and progression of a disease: a major aim of this work is to find new and effective therapeutic targets. Another expanding field of interest is the search for ncRNAs batteries as noninvasive diagnostic (i.e., liquid biopsies), prognostic, and predictive biomarkers. M. Barchitta and colleagues comprehensively review the role of miRNAs as potential biomarkers for adverse pregnancy outcomes (i.e., preeclampsia, spontaneous abortion, or preterm birth) and prenatal environmental exposure; M. Celano and colleagues focus on the possible use of miRNAs as sensitive and specific biomarkers for diagnosis and prognosis of thyroid cancers. If miRNAs likely are among the best functionally characterized ncRNAs, lncRNAs are the most represented RNA species in eukaryotic cells. Accordingly, the scientific community is making a great effort to functionally characterize these molecules, most of which are to date *orphan of function*. S. C. Credendino and colleagues have identified new isoforms of lncRNA Klhl14-AS, assaying their expression in a panel of mouse tissues and paving the way to their functional characterization. A. Matjašič and colleagues deal with the exploitation of lncRNAs as potential biomarkers for a detailed classification of gliomas by integrating their aberrant expression with that of miRNAs. A. Bolha and colleagues review the literature on the recently discovered circRNAs, by focusing on what is known about their involvement in cancer etiology and their use as effective biomarkers. It was becoming evident that cell networks cannot be appropriately investigated without considering the interplay between coding and noncoding RNAs. As reviewed by M. Ragusa and colleagues, the main challenge of ncRNA research in the next few years will be to unravel how ncRNAs can regulate each other in a cell type-specific biomolecular context and how this intricate interactions contribute to the final cell phenotype. A final and very important consideration is that we are firmly convinced that all present and future improvements (both theoretical and technological) of the scientific community should be considered in light of bioethics: the thirst for knowledge is one of the most sublime characteristics of man, but at its foundation there is always an ethical motivation. In today's scientific research, the experiment, in itself, can modify irreversibly what we are together with all that surrounds us (see CRIPSR/Cas9 technology). We must therefore be able to answer the following question: can genetic self-transformation be considered a legitimate means to free ourselves and increase individual autonomy? We also must ask ourselves if this aspect of liberal genetics may become something that could compromise individuals' self-understanding. As H. Jonas has revealed, “The value of all values is the possibility of value” [7].
